# Intellectual disability and antipsychotics

**DOI:** 10.1192/j.eurpsy.2021.1030

**Published:** 2021-08-13

**Authors:** I. Cuevas Iñiguez, M.D.C. Molina Lietor

**Affiliations:** Psiquiatría, Hospital Universitario Príncipe de Asturias, Alcalá de Henares, Spain

**Keywords:** intellectual disability, Antipsychotics

## Abstract

**Introduction:**

Intellectual disability is a condition of cognitive impairment and deficit in adaptive skills. Mental illness is frequent in people with intellectual disability. As a result antipsychotics are often prescribed to treat not only mental illness but also problem behaviors.

**Objectives:**

Perform a literature search about intellectual disability and antipsychotics.

**Methods:**

A non-systematic literature review was performed on PubMed using the keywords “intellectual disability” and “antipsychotics”. All papers published between 2015 and 2020 were evaluated.

**Results:**

A review of the literature reveals that antipsychotics are the most frequently prescribed psychotropic drugs in people with intellectual disability. However, results from the studies are ambiguous. Several studies showed that antipsychotics are effective in improving problem behaviours, nevertheless some recent studies showed no significant difference in the outcomes between antipsychotics and placebo

**Conclusions:**

Even though antipsychotics are prescribed in people with intellectual disability, evidence to support their use is lacking. In consequence, clinicians should consider the pharmacological approach as a part of an integrative treatment. Assessing adverse events, drug effects and the possibility of decreasing dose of antipsychotics is crucial.

